# Psychological well-being of identity-release egg donation parents with infants

**DOI:** 10.1093/humrep/dez201

**Published:** 2019-11-04

**Authors:** S Imrie, V Jadva, S Golombok

**Affiliations:** Centre for Family Research, University of Cambridge, UK

**Keywords:** egg donation, identity-release, parental psychological health, relationship quality, social support, assisted reproductive technologies, gamete donation

## Abstract

**STUDY QUESTION:**

What are the psychological health, relationship quality and perceived social support outcomes of heterosexual couples who have conceived an infant through identity-release egg donation?

**SUMMARY ANSWER:**

Parents’ scores on all measures were within the normal range. Egg donation mothers had poorer perceived social support, and egg donation fathers had less optimal psychological health than a comparison group of IVF parents, although these differences were associated with the older age of egg donation parents, rather than being an effect of family type.

**WHAT IS KNOWN ALREADY:**

There is limited understanding of the psychological health and couple relationship quality of egg donation parents, and no empirical data on parents’ social support, during the first year of parenthood. No studies have included families who have used an identity-release egg donor. The study offers the first examination of the psychological well-being of identity-release egg donation parents.

**STUDY DESIGN, SIZE, DURATION:**

This study included 57 families created through identity-release egg donation, and a comparison group of 56 families who had used IVF with their own gametes, recruited through UK fertility clinics. Families were visited at home between October 2013 and June 2015. The sample forms part of a larger study examining family functioning in families created following fertility treatment.

**PARTICIPANTS/MATERIALS, SETTING, METHOD:**

All families were heterosexual two-parent families with an infant aged 6–18 months. Mothers and fathers were administered standardised questionnaires assessing psychological health (Edinburgh Postnatal Depression Scale, Trait Anxiety Inventory and Parenting Stress Index-short form), couple relationship quality (Golombok Rust Inventory of Marital State) and perceived social support (Multidimensional Scale of Perceived Social Support).

**MAIN RESULTS AND THE ROLE OF CHANCE:**

Scores from the egg donation and IVF parents were within the normal range on all measures. Significant differences were found between the groups indicating less optimal social support in egg donation mothers compared to IVF mothers, and poorer psychological health in egg donation fathers compared to IVF fathers. These differences appeared to be related to the older age of egg donation parents or to twin parenthood, rather than to egg donation *per se*. No differences were found between the groups in the parents’ relationship quality.

**LIMITATIONS, REASONS FOR CAUTION:**

It is possible that families who were managing the transition to parenthood less well may have been less likely to participate in research. Fewer IVF than egg donation fathers participated in the study, so the statistical power was lower for comparisons between fathers.

**WIDER IMPLICATIONS OF THE FINDINGS:**

The findings are of relevance to UK clinics offering identity-release egg donation. That scores of egg donation parents on measures of psychological well-being were more similar than different to those of IVF parents should prove reassuring to individuals considering this treatment type. As less optimal outcomes were found for egg donation parents on several measures, and these were associated with parental age rather than conception type, it is recommended that clinics discuss with older patients how they may establish a social support network and signpost patients to appropriate post-natal support.

**STUDY FUNDING, COMPETING INTERESTS:**

This research was supported by a Wellcome Trust Senior Investigator Award [097857/Z/11/Z] and a CHESS-ESRC studentship. The authors have no conflicts of interest to declare.

**TRIAL REGISTRATION NUMBER:**

N/A

## Introduction

In 2017, over 4000 IVF cycles with donor eggs were carried out in the UK, two-thirds of which were undertaken by women aged 40 or over (Human Fertilisation and Embryology Authority, 2019). Little research has examined the psychological well-being of egg donation parents during the early years of their child’s life. The only study to do so compared 51 egg donation parents of infants (conceived using anonymous/known donation) to 80 natural conception parents, and found no differences between groups in mothers’ or fathers’ parenting stress, depression, anxiety or relationship satisfaction (Golombok *et al.*, 2004). (Note: ‘Egg donation parent’ is used throughout to refer to parents who have conceived a child through IVF/ICSI with a donor egg.) However, since the removal of donor anonymity in the UK in 2005, most egg donation cycles involve identity-release donation where the donor is unknown to the recipient but any resultant child may access identifying donor information at age 18. Despite its use in the UK for over a decade, nothing is known about the psychological well-being of parents in families created through identity-release egg donation.

Parental postnatal psychological health is crucial to positive family functioning. Mothers and fathers are at increased risk of depression in the first postnatal year, and postnatal depression is associated with a range of impaired developmental outcomes for children (Sweeney and MacBeth, 2016; Murray *et al.*, 2018). Postnatal anxiety may be as significant a risk for family functioning and child outcomes as depressive symptoms (Field, 2018; Philpott *et al.*, 2019).

Parenting stress, experienced when there is a mismatch between the demands of parenthood and the perceived availability of psychological and family resources (Abidin, 1990), has also been found to be associated with child adjustment (e.g. Sparks *et al.*, 2012). The availability of emotional support is known to buffer parents from the negative effects of parenting stress (Parkes *et al.*, 2015). Couple relationship quality is also important for child outcomes (Reynolds *et al.*, 2014) and studies of the transition to parenthood have consistently found that new parents experience declines in the functioning of the couple relationship (Doss and Rhoades, 2017).

Although all new parents face challenges during the transition to parenthood (Doss and Rhoades, 2017), parenting following identity-release egg donation may make this transition more complex. Concerns about family functioning in egg donation families have focused primarily on the absence of a genetic relationship between mothers and children. Egg donation parents typically experience parenthood within cultures that prioritise narratives of genetically-related families (Kirkman, 2008) and stigma about non-genetic parenthood may be a concern for parents (Golombok *et al.*, 2004). Internalised stigma has been found to be related to depression in new adoptive parents (Goldberg *et al.*, 2011), and similar concerns may be relevant to new egg donation parents.

Identity-release donation may pose greater challenges to parents than anonymous donation. Some couples believe that anonymous egg donation creates explicit psychological and practical boundaries between the recipient family and the donor (Laruelle *et al.*, 2011); identity-release donation could be perceived as offering less clear boundaries, and thus seem a less secure and reassuring option. It is possible that a sense of threat or a perceived lack of clear boundaries may adversely affect parental psychological health.

Individuals who have used assisted reproduction transition to parenthood following a period of infertility and fertility treatment, both of which may be highly stressful experiences (Greil *et al.*, 2010). The only study of psychological health of egg recipients prior to treatment found that 33% were clinically depressed and 59% had high distress levels (Carter *et al.*, 2011). Findings have been somewhat inconclusive with regard to whether successful treatment alleviates negative emotional responses in the longer term (Repokari *et al.*, 2005; Redshaw *et al.*, 2007). For egg donation parents, who have typically had a longer route to parenthood, the psychological toll of fertility treatment may be high.

Parents conceiving through egg donation may also be older than both their naturally conceiving peers (Golombok, 2004) and IVF parents (Imrie *et al.*, 2019). Families headed by older parents may benefit from greater financial stability, psychological preparedness for parenthood and more mature psychological capacities (Mac Dougall *et al.*, 2012; Camberis *et al.*, 2014). Although advanced maternal age does not increase mothers’ risk of postnatal depression (McMahon *et al.*, 2015), older parents may experience stigma around older parenthood (Mac Dougall *et al.*, 2012) and lower social support (Bornstein *et al.*, 2006), both of which may adversely affect psychological health. Pregnant egg donation mothers have stated concerns about the effects of advanced maternal age on motherhood (Hershberger, 2007).

Parental psychological health in assisted reproduction families may also be influenced by twin parenthood. Parents of twins have higher symptoms of depression, anxiety and parenting stress, compared to parents of singletons (Wenze *et al.*, 2015). Whilst risks associated with multiple births are relevant to all IVF families, egg donation parents are likely to experience twin parenthood in combination with advanced parental age and thus may be at greater risk of psychological health problems.

Given the paucity of research on the psychological health of identity-release egg donation parents, and the challenges specific to this group during the transition to parenthood, this study aimed to examine the psychological well-being of identity-release egg donation parents of infants. A sample of identity-release egg donation parents was compared to parents who had conceived through IVF using their own gametes, in order to control for the experience of fertility treatment. As identity-release egg donation parents have to manage the transition to non-genetic parenthood with the prospect of their child learning the donor’s identity in the future, and are older than their IVF counterparts, it was hypothesised that they would show poorer psychological health, social support and couple relationship quality than the IVF families.

## Materials and Methods

### Participants

A group of 57 egg donation families (57 mothers and 52 fathers), and a comparison group of 56 IVF families (56 mothers and 37 fathers) who had conceived using their own gametes, participated in the study. Infants were aged 6–18 months. The sample forms part of a larger study examining family functioning in families created through fertility treatment. Heterosexual-couple families with a child born in the previous 3–12 months were recruited through UK fertility clinics. Families with twins were included because multiple births are a common outcome of IVF (HFEA, 2019). For the larger study, clinics contacted 419 families by letter, of whom 190 sent their contact details to the researchers (response rate = 45%) (for full details of recruitment procedure, see Imrie *et al.*, 2019).

There was a significant difference between groups in mothers’ age (*t*(111) = 7.16, *P* < 0.001) and fathers’ age (*t*(87) = 3.27, *P* = 0.002) (Table I). Egg donation mothers were older (*M* = 42.54) than IVF mothers (*M* = 36.89), and egg donation fathers were older (*M* = 43.39) than IVF fathers (*M* = 39.32). There was no difference between groups in mothers’ educational level, *χ*^2^(1) = 0.51, *P* = 0.48, fathers’ educational level, *χ*^2^(1) = 0.09, *P* = 0.76, or couples’ relationship length, *t*(111) = 0.55, *P* = 0.74. Egg donation infants (*M* = 11.19) were older than IVF infants (*M* = 9.61), *t*(111) = 4.00, *P* < 0.001. There were similar proportions of twins in each group, *χ*^2^(1) = 0.41, *P* = 0.52. Egg donation parents had undergone more IVF cycles (*M* = 3.18) than IVF parents (*M* = 1.89), *U* = 1052.50, *P* = 0.001.

**Table I:** Family sociodemographic and fertility treatment characteristics by family type.

	Egg donation (*N* = 57)		IVF (*N* = 56)		Independent samples *t*-test		
	*M*	SD	*M*	SD	*t*	*P*	*d*
Mother’s age (years)	42.54	4.17	36.89	4.22	7.16	<0.001	1.35
Child’s age (months)	11.19	2.25	9.61	1.95	4.00	<0.001	0.75
Relationship (years)	12.45	4.87	11.96	4.51	0.55	0.74	0.10
	**Egg donation (*N* = 52)**		**IVF (*N* = 37)**				
	***M***	**SD**	***M***	**SD**	***t***	***P***	***d***
Father’s age (years)	43.39	5.99	39.32	5.44	3.27	0.002	0.71
	**Egg donation (*N* = 57)**		**IVF (*N* = 56)**		**Chi-square**		
	***N (%)***		***N (%)***		***χ^2^***	***df***	***P***
*Multiple rate*							
Singleton	52 (91%)		49 (88%)		0.41	1	0.52
Twin pair	5 (9%)		7 (12%)				
*Mother’s education*							
School education	12 (21%)		15 (27%)		0.51	1	0.48
Higher education	45 (79%)		41 (73%)				
*Father’s education*							
School education	15 (30%)		10 (27%)		0.09	1	0.76
Higher education	35 (70%)		27 (73%)				
					**Mann-Whitney *U*-test**		
Fertility Treatment	***M***	**SD**	***M***	**SD**	***U***	***P***	***d***
No. IVF cycles to conceive child	3.18	2.30	1.89	1.20	1052.50	<0.001	0.70

### Procedure

Families were visited at home by one of two trained psychologists as part of the larger study. Written informed consent was obtained from parents. Each parent was administered standardised questionnaires assessing psychological health, perceived social support and couple relationship quality. Ethical approval was granted by the University of Cambridge Psychology Research Ethics Committee. Data were collected between October 2013 and June 2015.

### Measures

#### Edinburgh Postnatal Depression Scale

The Edinburgh Postnatal Depression Scale (EPDS; Cox *et al.*, 1987) was administered to assess levels of depression. The 10-item questionnaire produces a total score between 0 and 30, with higher scores indicating higher levels of depression. Items include ‘Things have been getting on top of me’. It has satisfactory validity and split-half reliability (Cox *et al.*, 1987) and has been validated for use with fathers in the postnatal period (Edmondson *et al.*, 2010). Cut-off scores of 13 and above indicate probable major depression in post-partum mothers (Gibson *et al.*, 2009) and scores of 11 and above indicate this in fathers (Edmondson *et al.*, 2010). Cronbach’s alphas for the sample were 0.84 (mothers) and 0.85 (fathers).

#### Trait Anxiety Inventory

Anxiety levels were assessed using the Trait Anxiety Inventory (TAI; Spielberger, 1983). Scores on the 20-item questionnaire range from 20 to 80. Higher scores reflect greater levels of anxiety. Items include ‘I feel rested’ and ‘I feel like a failure’. The questionnaire has good reliability and discriminates well between clinical and non-clinical groups (Spielberger, 1983). There is no standardised cut-off for the TAI (Glasheen *et al.*, 2010). Studies examining postnatal anxiety in mothers have often used cut-off scores of over 40 (Grant *et al.*, 2008) to indicate high anxiety. Cronbach’s alphas for mothers’ and fathers’ scale items were 0.87 and 0.90, respectively.

**Table II:** Correlations between variables.

Variable	1.	2.	3.	4.	5.	6.	7.	8.	9.	10.	11.	12.	13.	14.	15.
1. Mother age	-														
2. Father age	0.55^**^	-													
3. Child age	0.18	0.14	-												
4. Relationship	0.18	0.14	0.11	-											
5. IVF cycles	0.22^*^	0.18	0.14	0.33^**^	-										
6. EPDS (mother)	0.14	0.22^*^	0.05	−0.01	−0.13	-									
7. TAI (mother)	0.20^*^	0.22^*^	0.06	0.07	−0.11	0.78^**^	-								
8. PSI (mother)	0.28^*^	0.24^*^	0.04	0.03	−0.19^*^	0.50^**^	0.60^**^	-							
9. GRIMS (mother)	0.19^*^	0.32^*^	0.09	−0.05	−0.11	0.52^**^	0.45^**^	0.47^**^	-						
10. MSPSS (mother)	−0.34^**^	−0.35^*^	−0.03	−0.13	−0.04	−0.42^**^	−0.42^**^	−0.26^*^	−0.38^**^	-					
11. EPDS (father)		0.25^*^	0.07	0.07	−0.04	0.19	0.11	0.24^*^	0.16	−0.28^*^	-				
12. TAI (father)		0.16	0.15	0.04	−0.10	0.17	0.10	0.21	0.20	−0.24^*^	0.75^**^	-			
13. PSI (father)		−0.002	−0.10	0.05	−0.18	0.10	0.08	0.33^*^	0.03	−0.14	0.44^**^	0.57^**^	-		
14. GRIMS (father)		0.07	0.09	−0.02	−0.19	0.32^*^	0.23^*^	0.36^*^	0.58^**^	−0.19	0.27^*^	0.44^**^	0.34^*^	-	
15. MSPSS (father)		−0.22^^*^^	0.02	−0.11	−0.07	−0.25^*^	−0.29^*^	−0.24^*^	−0.46^**^	0.28^*^	−0.28^*^	−0.34^*^	−0.20	−0.48^**^	-

^*^ *P* < 0.05

^**^ *P* < 0.001

#### Parenting Stress Index

Parents were administered the short form of the Parenting Stress Index (PSI-SF; Abidin, 1990), to assess stress associated with parenting. The questionnaire comprises 36 items (e.g. ‘I feel trapped by my responsibilities as a parent’). Total scores range from 36 to 180. Higher scores reflect greater parenting stress. Concurrent and predictive validity have been demonstrated for the full-length questionnaire, and the short form correlates highly with the full-length version (Abidin, 1990). Cronbach’s alphas were 0.91 (mothers) and 0.89 (fathers).

#### Multidimensional Scale of Perceived Social Support

The Multidimensional Scale of Perceived Social Support (MSPSS; Zimet *et al.*, 1988) was administered to assess parents’ perceived social support. The 12-item questionnaire comprises three subscales measuring the perceived adequacy of support from family, friends and significant other. Each item (e.g. ‘My family really tries to help me’) is rated on a seven-point scale. Higher scores indicate higher perceived social support. The MSPSS has good validity and test–retest reliability (Zimet *et al.*, 1988). Mean scale scores of 1–2.9 can be classified as low support, 3–5 as moderate support and 5.1–7 as high support (Zimet *et al.*, 1988). Cronbach’s alphas for the present study were 0.95 (mothers) and 0.91 (fathers).

#### Golombok Rust Inventory of Marital State

Parents were administered the Golombok Rust Inventory of Marital State (GRIMS; Rust *et al.*, 1990), a 36-item questionnaire assessing the quality of the relationship between couples. Each item (e.g. ‘I am dissatisfied with our relationship’) is rated on a four-point scale. Scores range from 0 to 84. Higher scores indicate poorer relationship quality. A score >34 indicates relationship dissatisfaction. The measure discriminates significantly between couples who are about to separate and those who are not (Rust *et al.*, 1990). Cronbach’s alphas for mothers’ and fathers’ scale items were 0.92 and 0.90, respectively.

### Analytical Approach

Differences between egg donation and IVF families on outcome variables were assessed using multivariate analyses of variance (MANOVAs) and univariate analyses of variance (ANOVAs). Where multiple univariate ANOVAs were used, a Bonferroni correction was applied to the alpha level. Correlations between dependent variables were carried out to check for the presence of multicollinearity (Table II). Box’s tests were used to test for the assumption of homogeneity of variance–covariance matrices.

#### Twins

For data analysis, one twin was randomly selected. As parenting twins is known to affect parents’ psychological health (Wenze *et al.*, 2015), analyses were carried out twice, once with the full sample, and once with the singleton-only families.

#### Covariates

The groups differed in demographic (mother’s age, father’s age and number of children) and fertility treatment history variables (number of IVF cycles). These variables are related systematically to the defining characteristics of the groups (e.g. egg donation parents are known to be older parents (Golombok *et al.*, 2004). Miller and Chapman (2001) suggest that in such cases, potential covariates should be viewed as a substantial, meaningful part of the analysis, and not included as covariates. In cases where a significant difference in outcome variables was found between groups, and a significant relation existed between the outcome variable and a covariate, a covariate was used. In these instances, covariates were used to understand whether differences between groups could be explained by one of the covariates, or if they genuinely reflected an effect of family type.

Child age differed between egg donation and IVF families. Child age was not significantly associated with any of the outcome measures (Table II), so was not controlled for in the analyses.

## Results

### Mothers

#### Psychological health

A MANOVA including mothers’ scores for depression, anxiety and parenting stress found no significant difference between mothers in egg donation and IVF families (see Table III). Egg donation and IVF mothers did not differ in their psychological health.

**Table III:** Means, SD, ANOVA (F), significance levels (*P*), effect sizes (d) and 95% CI values for comparisons of mothers’ psychological health, relationship satisfaction and perceived social support between family types.

	Egg donation		IVF		*F*	*P*	*d*	95% CI
	(*N* = 54)		(*N* = 56)		(1, 108)			
	*M*	SD	*M*	SD				
EPDS	5.76	4.69	5.16	3.59				
TAI	36.78	9.75	34.70	7.57	0.58	0.63		
PSI	60.76	15.14	59.37	12.85				
GRIMS^1^	23.54	11.83	21.37	10.37	1.00	0.32	0.20	[−0.18, 0.57]
	**Egg donation**		**IVF**		***F***	***P***	***d***	**95% CI**
	**(*N* = 57)**		**(*N* = 54)**		**(1, 109)**			
	***M***	**SD**	***M***	**SD**				
MSPSS								
Total	5.63	1.25	6.28	0.77	10.83	<0.001	−0.53	[−0.91, −0.15]
Family	5.26	1.54	6.17	0.95	13.89	<0.001	−0.71	[−1.09, −0.32]
Sig. other	6.02	1.33	6.57	0.91	6.52	0.01	−0.48	[−0.86, −0.10]
Friends	5.60	1.28	6.05	1.22	3.60	0.06	−0.36	[−0.74, 0.02]

^1^ED *N* = 56, IVF *N* = 51

There were five (9%) egg donation and two (4%) IVF mothers who scored at or above the clinical cut-off point for probable depression. For anxiety, 14 (25%) egg donation and 10 (18%) IVF mothers scored over the cut-off point of 40.

#### Relationship satisfaction

A one-way ANOVA test of mothers’ relationship satisfaction scores was non-significant (Table III). There were 39 (70%) egg donation mothers and 41 (80%) IVF mothers who scored 29 or less, indicating above-average relationship quality. There were six (11%) egg donation and two (4%) IVF mothers who scored 42 or above, indicating severe problems in the relationship.

#### Perceived social support

A one-way ANOVA test of mothers’ total scores for perceived social support found a significant different between groups, *F*(1, 109) = 10.83, *P* < 0.001. Egg donation mothers perceived lower levels of social support than IVF mothers (Table III).

The MSPSS subscales were examined individually using univariate ANOVAs. The ANOVA for the family subscale was significant at the adjusted alpha level of 0.02, *F*(1, 110) = 13.89, *P* < 0.001, with egg donation mothers perceiving less family support than IVF mothers. The effect size was medium to large (*d* = −0.71). The ANOVA for the significant other subscale was also significant at the adjusted alpha level, *F*(1, 110) = 6.52, *P* = 0.01. Egg donation mothers perceived lower levels of social support from a significant other than IVF mothers. The effect size was medium (*d* = −0.48). The ANOVA for the friends subscale was non-significant at the adjusted alpha level, *F*(1, 111) = 3.60, *P* = 0.06. The effect size was small to medium (*d* = −0.36).

When mother’s age was included as a covariate, the test for total perceived social support was no longer significant, *F*(1, 108) = 2.06, *P* = 0.15, and neither were the ANCOVAs for the subscales at the adjusted alpha level (family *F*(1, 109) = 3.06, *P* = 0.08, significant other *F*(1, 109) = 1.89, *P* = 0.17 and friends *F*(1, 110) = 0.17, *P* = 0.68). This indicates that the lower perceived social support of egg donation mothers was associated with their older age.

There were 44 (77%) egg donation and 51 (94%) IVF mothers who had mean total scores that represented high perceived social support. Eleven (19%) egg donation and 3 (6%) IVF mothers had moderate social support, whilst 2 (4%) egg donation mothers had low perceived social support.

#### Singleton-only families

None of the findings changed when the analyses were rerun without the data from the mothers of twins (singleton-only sample: 52 egg donation, 49 IVF).

### Fathers

#### Psychological health

A MANOVA including fathers’ scores for depression, anxiety and parenting stress found a significant difference between egg donation and IVF fathers for fathers’ psychological health, *F*(1, 81) = 2.93, *P* = 0.04 (Table IV).

**Table IV:** Means, SD, ANOVA (F), significance levels (*P*), effect sizes (d) and 95% CI values for comparisons of fathers’ psychological health, relationship satisfaction and perceived social support between family types.

	Egg donation		IVF		*F*	*P*	*d*	*F*	*P*	*d*	95% CI
	(*N* = 49)		(*N* = 34)		(1, 81)						
	*M*	SD	*M*	SD							
EPDS	5.51	4.07	3.47	3.16				6.01	0.02	0.55	[0.10, 0.99]
TAI	35.04	8.60	32.76	6.13	2.93	0.04		1.76	0.19	0.30	[−0.14, 0.074]
PSI	57.88	12.10	58.68	12.66				0.09	0.77	−0.07	[−0.50, 0.37]
	**Egg donation**		**IVF**		***F***	***P***	***d***				**95% CI**
	**(*N* = 51)**		**(*N* = 37)**		**(1, 86)**						
	***M***	**SD**	***M***	**SD**							
GRIMS	19.16	9.97	20.73	10.19	0.52	0.47	−0.16				[−0.58, 0.27]
MSPSS											
Total^1^	5.72	0.87	5.83	0.84	0.36	0.55	−0.13				[−0.55, 0.29]
Family^1^	5.52	1.23	5.57	1.15	0.03	0.87	−0.04				[−0.46, 0.38]
Sig. other^1^	6.33	0.87	6.34	0.83	0.001	0.97	−0.01				[−0.43, 0.41]
Friends^1^	5.30	1.12	5.57	1.08	1.31	0.26	−0.25				[−0.67, 0.18]

^1^ED *N* = 52

The univariate test for the EPDS was significant at the adjusted alpha level of 0.02, *F*(1, 81) = 6.01, *P* = 0.02, with egg donation fathers showing higher levels of depression than IVF fathers. The effect size was medium (*d* = 0.55). No differences were found between groups on the TAI, *F*(1, 81) = 1.76, *P* = 0.19, or the PSI, *F*(1, 81) = 0.09, *P* = 0.77.

When father’s age was included as a covariate, the MANCOVA for psychological health scores was no longer significant, *F*(1, 80) = 1.77, *P* = 0.16. When the univariate tests were examined, the EPDS was not significant at the adjusted alpha level of 0.02, *F*(1, 80) = 3.34, *P* = 0.07. This indicated that the higher depression scores of egg donation fathers appeared to be associated with their older age rather than being an effect of family type.

As with mothers’ scores, most of the fathers’ scores fell within the normal range. There were 8 (15%) egg donation fathers who scored higher than the cut-off point for probable depression, whilst 15 (29%) egg donation and 4 (11%) IVF fathers had scores indicating high anxiety.

#### Relationship satisfaction

A one-way ANOVA examining fathers’ GRIMS scores was not significant. Fathers in egg donation and IVF families did not differ in their relationship satisfaction (Table IV). There were 41 (80%) egg donation and 31 (84%) IVF fathers who had above average relationship satisfaction. Only one (2%) egg donation and two (5%) IVF fathers scored 42 or above, indicating severe relationship problems.

#### Perceived social support

A one-way ANOVA examining fathers’ MSPSS total scores was not significant (Table IV), indicating no difference in levels of perceived social support between egg donation and IVF fathers.

There were 41 (79%) egg donation and 32 (86%) IVF fathers who had mean scores indicating high social support, whilst 11 (21%) egg donation and 5 (14%) IVF fathers had moderate social support.

#### Singleton-only families

The MANOVA examining psychological health found no significant difference between egg donation and IVF fathers, *F*(1, 70) = 2.02, *P* = 0.12 (singleton-only sample: 47 egg donation, 31 IVF).

## Discussion

Psychological health, couple relationship quality and perceived social support were examined in families created through identity-release egg donation, and a comparison group of families who had conceived through IVF. Risk factors associated with difficulties in family functioning (couple relationship problems and parental psychopathology) were absent in both family types and scores on all measures were within the normal range. Significant differences were found between groups indicating less optimal social support amongst egg donation mothers and poorer psychological health amongst egg donation fathers. These differences appeared to be related to the older age of egg donation parents rather than egg donation *per se*.

It was hypothesised that egg donation mothers would show greater psychological problems, given concerns about the effect of identity-release donation on parents’ psychological wellbeing, and in line with suggestions that women may have high levels of psychopathology prior to egg donation treatment (Carter *et al.*, 2011); this hypothesis was not supported. Mothers in egg donation and IVF families were psychologically well-adjusted, with levels of postnatal depression and parenting stress within the normal range. Although some studies have found that mothers conceiving through fertility treatment experience ongoing psychological distress (Redshaw *et al.*, 2007), the current findings are more in line with studies showing that parents conceiving through fertility treatment may be particularly psychologically resilient (Repokari *et al.*, 2005).

In line with the study hypothesis, egg donation fathers showed higher levels of depression than IVF fathers. Eight egg donation fathers scored above the clinical cut-off point for probable depression, equating to 15% of the egg donation sample. This is slightly higher than the 10.4% rate of paternal depression between the first trimester and 1 year post-partum in the general population (Paulson and Bazemore, 2010). When factors associated with the higher scores of egg donation fathers were explored, the older age of egg donation fathers explained their higher levels of depression. Whilst higher depressive symptoms have been found in older ART fathers of 4–11-year-olds (Boivin *et al.*, 2009), little research exists on the postnatal psychological health of either older fathers or older ART fathers, and both merit further investigation.

Interestingly, when comparisons between groups were rerun without data from twin families, no differences were found between the groups for psychological health of fathers, suggesting that the difference found between egg donation and IVF fathers in the full sample may be explained by poorer psychological health amongst egg donation fathers of twins. Fathers of twins have been found to have elevated levels of post-natal depression (Wenze *et al.*, 2015), and it is possible that twin parenthood, combined with the older age of egg donation fathers, contributed to their higher depression scores, although these findings require replication with larger samples. It is also probable, however, that any small to medium differences would not have been detected with the smaller singleton-only sample due to lower statistical power.

Around one-quarter of egg donation mothers and almost one-third of egg donation fathers scored above the cut-off score for anxiety. Studies examining postnatal anxiety have reported prevalence rates of 3–43% (Fallon *et al.*, 2016), and because of the variability of studies, it is hard to determine an average prevalence (Field, 2018). As a result, it is difficult to establish the extent to which the current sample’s anxiety rates compare to normative samples. The prevalence of high anxiety in new ART parents may be worthy of further exploration to establish those most at risk.

No differences were found between egg donation and IVF families for either mothers’ or fathers’ ratings of their relationship. Both family types showed high quality couple relationships, with most parents in both groups obtaining scores indicating above average relationship quality. This finding adds to the small body of literature that found high relationship satisfaction in parents of ART infants (Golombok *et al.*, 2004; Sydsjö *et al.*, 2002). Despite normative declines in couple relationship quality over the transition to parenthood, several longitudinal studies have found that a minority of parents reported stability in relationship functioning (Doss and Rhoades, 2017). Couples who persist with fertility treatment may comprise a self-selected group with strong coping skills (Repokari *et al.*, 2007), which may enable egg donation and IVF parents to manage the transition to parenthood well.

Egg donation mothers reported lower perceived social support from family than did IVF mothers, which was associated with their older age, rather than being an effect of family type. The findings are consistent with research showing that maternal age was associated with less support from extended family amongst mothers of 5-month-olds (Bornstein *et al.*, 2006) and amongst adoptive parents (McKay and Ross, 2010). Given that older parents are likely to have parents who are themselves older, it is perhaps not surprising that they may perceive support as less available. Egg donation mothers did, however, feel supported by their friendship groups, and fathers felt that their support needs were well met.

A limitation of the current study is that it is not possible to ascertain whether families who participated differed systematically from those who declined participation. It is possible that families who did not feel they were managing new parenthood well, or who did not want to discuss egg donation, may have been less likely to participate and may differ from responders in their psychological well-being.

The analyses are also limited by the number of fathers who took part in the study, with fewer IVF than egg donation fathers participating. Statistical power was thus lower for comparisons between egg donation and IVF fathers, although the sample size was sufficient to detect a large effect at *α* = 0.05 (Cohen, 1992). Future studies should attempt to recruit larger samples to ensure that there is sufficient power to detect small to medium effect sizes between groups. Fathers remain under-represented in family research (Cabrera *et al.*, 2018), and having a thorough understanding of both parents’ psychological health in two-parent families is crucial for understanding how the family system functions in infancy, particularly given that parents’ psychological health outcomes are often related (Paulson and Bazemore, 2010). The findings are limited in their generalisability to other sociocultural contexts, given the homogeneity of the sample’s ethnicity and educational level, and that all families lived in the United Kingdom.

Notwithstanding these limitations, the sample represents the only sample to date assessing the psychological well-being of identity-release egg donation families and is the only study to control for experiences of fertility treatment when examining the psychological health of egg donation parents with infants. Previous research into identity-release donation had focused on sperm donation families (e.g. Golombok *et al.*, 2016), with little known about identity-release egg donation families in the UK, despite it being the main treatment option for over a decade. The study also offers the only quantitative assessment to date of egg donation parents’ perceived social support, a construct crucial to understanding parental psychological health yet understudied in assisted reproduction samples.

As the first examination of psychological well-being in families created through identity-release egg donation, the findings are of relevance to fertility clinics in the UK. The finding that parents in both family types showed good psychological health outcomes, and that egg donation parents showed more similarities than differences to IVF parents, should prove reassuring to clinicians and families considering the treatment options.

Clinics and prospective parents should be aware that where differences were found, specifically, poorer psychological health amongst egg donation fathers and poorer social support amongst egg donation mothers, these were associated with parental age. It may be that discussions with patients about the importance of establishing social support networks prior to birth, or providing information about common signs of post-natal mental health problems and how to access support, could be incorporated into pre- or post-treatment clinical contact. As men are less likely to access support for psychological health problems than are women (Vogel *et al.*, 2014), a more open discussion of paternal postnatal psychological health may be beneficial. The differences found between groups, however, should not be a cause for concern, as the parents’ scores on all measures in both family types were within the normal range.

## Acknowledgements

We would like to thank all the families who took part in this study and Sarah Evans who provided research assistance. We also thank CARE Fertility, Bourn Hall Fertility Clinic, The London Women’s Clinic, Bath Fertility Centre, Oxford Fertility, Edinburgh Fertility and Reproductive Endocrine Centre, GCRM, Wessex Fertility, and Aberdeen Fertility Centre.

## Authors’ roles

All authors contributed to the study design and interpretation of data for this study. This manuscript was drafted by SI and has been approved by all authors.

## Funding

This research was supported by a Wellcome Trust Senior Investigator Award [097857/Z/11/Z] and a CHESS-ESRC studentship.

## Conflict of interest

The authors have no conflicts of interest to declare.
